# Protein Under-Wrapping Causes Dosage Sensitivity and Decreases Gene Duplicability

**DOI:** 10.1371/journal.pgen.0040011

**Published:** 2008-01-18

**Authors:** Han Liang, Kristina Rogale Plazonic, Jianping Chen, Wen-Hsiung Li, Ariel Fernández

**Affiliations:** 1 Department of Ecology and Evolution, University of Chicago, Chicago, Illinois, United States of America; 2 Program in Applied and Computational Mathematics, Princeton University, Princeton, New Jersey, United States of America; 3 Program in Applied Physics, Rice University, Houston, Texas, United States of America; 4 Department of Bioengineering, Rice University, Houston, Texas, United States of America; 5 Department of Computer Science, University of Chicago, Chicago, Illinois, United States of America; University of Aarhus, Denmark

## Abstract

A fundamental issue in molecular evolution is how to identify the evolutionary forces that determine the fate of duplicated genes. The dosage balance hypothesis has been invoked to explain gene duplication patterns at the genomic level under the premise that a dosage imbalance among protein-complex subunits or interacting partners is often deleterious. Here we examine this hypothesis by investigating the molecular basis of dosage sensitivity. We focus on the extent of protein wrapping, which indicates how strongly the structural integrity of a protein relies on its interactive context. From this perspective, we predict that the duplicates of a highly under-wrapped protein or protein subunit should (1) be more sensitive to dosage imbalance and be less likely to be retained and (2) be more likely to survive from a whole-genome duplication (WGD) than from a non-WGD because a WGD causes little or no dosage imbalance. Our under-wrapping analysis of more than 12,000 protein structures strongly supports these predictions and further reveals that the effect of dosage sensitivity on gene duplicability decreases with increasing organismal complexity.

## Author Summary

A gene duplication provides an extra gene copy that can be free to accumulate mutations and gain a new function. Therefore, gene duplication plays a very important role in evolution. However, the presence of an additional gene copy can sometimes be deleterious because it can lead to an excessive dosage relative to those of its interacting partners. This dosage imbalance effect in turn influences the fate of duplicated genes in evolution. Our study gives the first description to our knowledge of the molecular/structural basis for the dosage imbalance effect. We study the relationships between gene family size and extent of protein under-wrapping, a molecular quantifier of the reliance of the protein on binding partnerships to maintain structural integrity, indicative of the extent of structure protection from disruptive hydration. Using more than 12,000 protein three-dimensional structures from six organisms that range from bacteria to human, we show an inverse relationship between extent of protein under-wrapping and family size. That is, a duplication is unlikely to be tolerated if the protein is highly under-wrapped (i.e., its structure requires substantial stabilizing interactions with other proteins). We also show that the effect of dosage imbalance is more apparent in unicellular organisms but is buffered to some extent in higher eukaryotes.

## Introduction

Gene duplication is a primary source for the emergence of new genes and increases genome complexity [1,2]. In recent years, the evolutionary forces influencing gene duplicability have been under intense study. In particular, the gene dosage balance hypothesis [3] has been often invoked to explain gene duplication patterns at the genomic level [4]. The dosage balance hypothesis states that an imbalance in the concentrations of the subcomponents of macromolecular complexes can be deleterious [3]. Although this notion was originally proposed in the context of protein complexes, it can be extended to other protein interaction partnerships [5]. If dosage imbalance is indeed deleterious, the outcome of a gene duplication event would largely depend on the immediate dosage sensitivity effect. While significant progress has been made in the last several years [4,6–9], the influence of dosage imbalance on the retention of gene duplicates remains not well understood. So far, the most relevant studies on this topic have mainly focused on protein complex data or protein-protein interaction data, which have inherent limitations. First of all, such data represent the interacting context of a protein in an abstract way. For example, the potential dosage imbalance effect of protein subunits in a complex may crucially depend on their topological positions within the complex and on the complex-assembly pathway [5]. Second, more importantly, there is a conceptual distinction between a-priori plausible protein associations and obligatory associations required to preserve the structural integrity and functionality of the protein. Thus, even if the interacting context of a protein could be characterized by some measurements (e.g., protein connectivity or interacting surface), the potential imbalance effect would still be hard to assess. Lastly, it is known that most current protein interaction data are noisy, being plagued with both false positives and false negatives [10,11].

Recent advances in structural genomics and biophysics enable us to examine the dosage balance hypothesis in the light of the three-dimensional structure of proteins. In this regard, we focus on a specific attribute of protein structure, the so-called under-wrapping [12–17]. This attribute quantifies the extent to which the protein structure is reliant on the interactive context to maintain its integrity. In particular, overexpressing a highly under-wrapped protein can increase the propensity for aberrant misfolding and aggregation [16], promoting dosage sensitivity.

**Figure 1:**
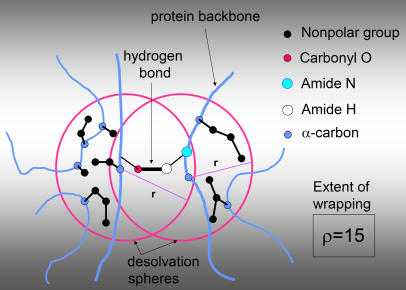
Protein Under-Wrapping The extent of wrapping of a single intramolecular hydrogen bond. This parameter defines the solvent-exposure extent of the bond. The hydrogen bond is mainly an electrostatic interaction between opposite partial charges in the amide and carbonyl groups of the paired residues. A desolvation domain defines the local microenvironment of the hydrogen bond and is depicted as the union of two spheres centered at the α-carbons of the paired residues. The outer boundaries of the desolvation balls are indicated by magenta circles. The solid black disks represent non-polar carbonaceous groups on the residue side chains. These non-polar groups “wrap” the bond by excluding surrounding water, thereby protecting the structure from the competing hydration of the polar amide and carbonyl groups. The solid blue dots represent the α-carbons on the protein backbone, which in turn is depicted by curved blue lines. The extent of wrapping (ρ) is defined as the number of non-polar groups in the desolvation domain. Thus, an under-wrapped hydrogen bond, or *dehydron*, is one whose wrapping is insufficient, as statistically defined in Methods.

The under-wrapping parameter describes the solvent accessibility of the major determinants of protein structure: the backbone hydrogen bonds (Figure 1). Thus, in order for the structure to prevail and remain functionally competent, backbone hydrogen bonds must be “wrapped” by clusters of non-polar amino acid residues that exclude the surrounding water, thereby preventing the competing hydration of the paired polar groups. Since backbone hydration competes with structure retention, the intramolecular hydrogen bonds that are water-accessible, termed *dehydrons* [13], represent structural vulnerabilities. As a consequence, dehydrons promote binding partnerships with the concurrent exclusion of surrounding water, as needed to maintain the structural integrity of the protein [13,15,17]. The hydrogen-bond protection requirement poses a strong constraint on protein architecture and dictates that highly under-wrapped proteins, i.e., those with a large number of dehydrons, should be highly interactive [15] to maintain their structural integrity.

As shown in Figure 1, the wrapping extent can be accurately determined from reported structure deposited in the Protein Data Bank (PDB) [12]. As protein structures become more under-wrapped, they become more reliant on binding partnerships [15]. Thus, protein under-wrapping quantifies how strongly the structural integrity of a protein depends on its binding partners [13], thereby framing a vantage point to study the dosage imbalance effect.

## Results/Discussion

**Figure 2:**
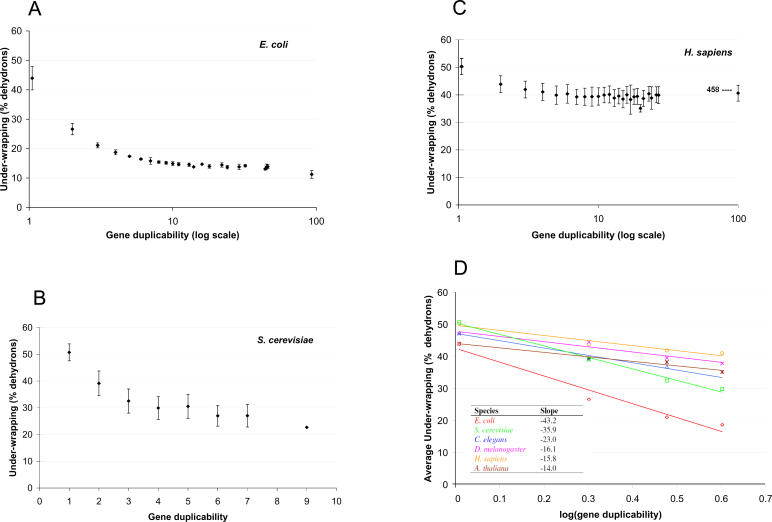
Negative Correlations between Protein Under-Wrapping Extent and Gene Duplicability In E. coli (A), in yeast (B), in human (C), and in slopes in six organisms (D). Here gene duplicability is defined as the gene family size (*m*). Because of the huge spread in duplicability for E. coli and H. sapiens, a log scale was adopted on the abscissas for (A) and (C). The mean extent of wrapping is determined by averaging over all genes binned by gene duplicability value. The error bars indicate ± a standard deviation from the mean values. The slopes in (D) are determined by the least squares linear regression from *m* = 1 to 4. The under-wrapping data are provided in Tables S1–S6.

From the above reasoning we predict that the probability of retention of gene duplicates in evolution (i.e., gene duplicability) should decrease with the extent of hydrogen bond under-wrapping of the polypeptide encoded by the gene. To test this prediction, we compiled non-redundant proteins with PDB-reported structures, calculated the under-wrapping extent for each protein (subunit), and determined the duplicability (*m*, the gene family size) for the corresponding gene. Interestingly, in all six organisms studied (Escherichia coli, yeast, worm, fly, human and thale cress), we found a negative correlation between protein under-wrapping extent and gene duplicability (Figures 2A–2C and S1).

Since it has been shown that genes with particular biological functions tend to duplicate in evolution [18–20], we examined the potential influence of functional bias on our results. We compared the under-wrapping extent of yeast singletons with that of duplicates in different functional categories and found that singletons are consistently more under-wrapped than duplicates in each functional category (Figure S2). This result indicates that the effect of protein wrapping on gene duplicability is independent from the previously known functional bias of gene duplication.

**Figure 3:**
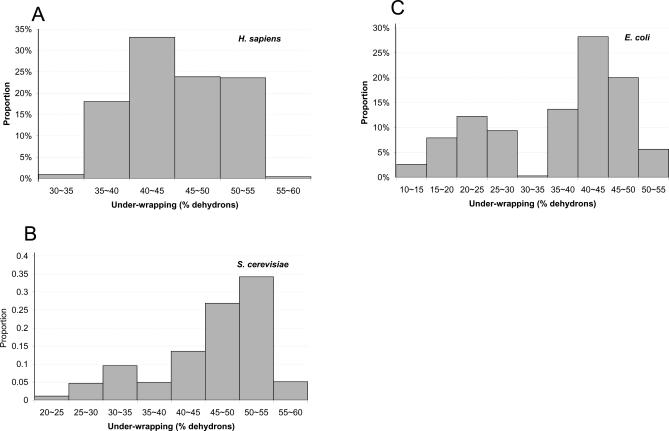
The Distributions of Per-Gene-Family Protein Under-Wrapping in Human (A), in Yeast (B), and in E. coli (C) The abscissa indicates the bins of the percentages of dehydrons over the total number of hydrogen bonds in the protein.

Our study reveals a universal negative effect of protein under-wrapping on gene duplicability in a variety of species, strongly supporting the dosage balance hypothesis. The decreasing tendency is most significant from *m* = 1 to 4 and becomes less obvious at higher duplicability. However, the dependence between the two variables in different species varies a lot: the negative correlation is quite strong in simple organisms such as E. coli and yeast, but becomes weak in complex organisms such as humans. To perform a more rigorous comparison, we used the linear regression to roughly capture the dependence between protein under-wrapping and gene duplicability. As shown in Figure 2D, as organismal complexity increases, the effect of protein under-wrapping on gene duplicability decreases, that is, E. coli > yeast > worm > fly ∼ human ∼ thale cress, suggesting a less important role of the dosage imbalance effect in complex organisms. To further understand this intriguing trend, we examined the per-gene-family protein under-wrapping distributions in different species. As shown in Figure 3, E. coli and yeast proteins have relatively broad under-wrapping distributions, while human proteins show a narrow distribution mainly from 35% to 55%. There are fewer well-wrapped proteins (<35%) in humans, implying that most human proteins need binding partners to maintain the integrity of their functional structure. On the other hand, unicellular species appear to possess more autonomous protein folders (under-wrapping <35%), capable of operating without forming obligatory complexes [17]. However, the contrasting distributions between complex and simple organisms are hard to interpret, due to the staggering difference at the proteome level.

**Figure 4:**
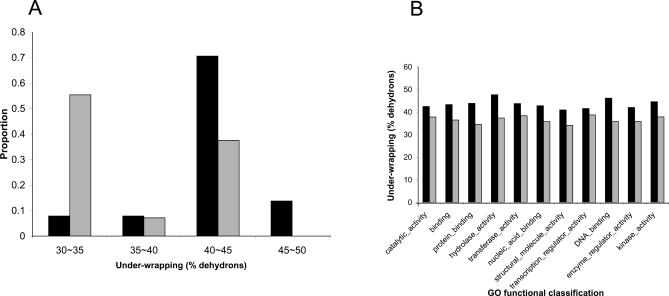
Contrasting Protein Under-Wrapping Patterns between Yeast WGD and Non-WGD Duplicates (A) The under-wrapping distributions between the two groups. The abscissa indicates the bins of the percentages of dehydrons over the total number of hydrogen bonds in the protein. (B) Average under-wrapping values in different functional categories. In both panels, black bars represent WGD duplicates and gray bars represent non-WGD duplicates.

Duplicated genes can arise from either whole-genome duplication (WGD) or non-WGD (including individual or segmental duplication) [21]. In a WGD, every gene in the genome is duplicated at the same time, so that binding partnerships are also duplicated, leading to less chance of dosage imbalance than a non-WGD. Thus, an interesting prediction stemming from the dosage balance hypothesis is that duplicates of highly under-wrapped proteins would be more likely to survive from a WGD than from a non-WGD event. Since the duplication history of yeast genes has been largely elucidated [22], we decided to test this prediction using yeast duplicates with *m* = 2. We classified the yeast duplicates into two groups: one group from WGD and the other from non-WGD. By performing the analysis conditioned on the same *m*, the under-wrapping difference between the two groups should mainly be determined by the underlying duplication mechanisms. We found that the under-wrapping extent in WGD duplicates is significantly higher than that in non-WGD duplicates (Figure 4A, *N*_WGD_ = 51, *N*_non-WGD_ = 56, two-tailed Wilcox rank test *p* < 8 × 10^−10^), implying that the dosage imbalance effect was indeed relaxed in the WGD. Again, we examined this trend in different functional categories and found that the WGD duplicates are consistently more under-wrapped than the non-WGD duplicates in each category (Figure 4B).

In higher eukaryotes, considerable amount of highly under-wrapped proteins are associated with highly duplicated genes, suggesting that complex organisms are less sensitive to the dosage imbalance effect. This can possibly be attributed to several factors. First, complex organisms may have more efficient systems to adjust gene expression levels (e.g., chaperons, proteases and non-coding RNAs). It has been shown that in cultured cells more than 60% human promoter polymorphisms cause more than two-fold differences in gene-expression level [23]. Second, widespread alternative splicing in higher eukaryotes may play an important role to fix the imbalance effect, since different splicing variants might represent an “escape route” to avoid dosage imbalance. Third, it has been suggested that proteins tend to physically interact with similar partners, especially with their own duplicates [24]. Complex organisms may have higher allostery (i.e., dimerization or oligomerization), which can partly alleviate dosage imbalance. Fourth, complex organisms generally have a smaller effective population size than do simple organisms [25], so that a duplicate bearing a slightly deleterious dosage imbalance effect would have a better chance to be fixed in the population, thereby allowing a longer time for functional innovation. Last but no the least, adaptation (positive selection) due to functional diversification may have played an important role in determining the retention of duplicated genes in complex organisms [26,27] (e.g., MHC genes in mammals [28]).

In summary, we have identified protein under-wrapping as a molecular basis of dosage sensitivity. An imbalance-generating duplication becomes less tolerable if the protein is severely under-wrapped and therefore requires substantial stabilizing interactions with other proteins. Indeed, the extent of under-wrapping in a protein can be used as an approximate predictor of the strength of the effect of dosage imbalance on gene duplicability. The prediction can be made more broadly and precisely in the future when more data on protein structures, especially on protein complexes, become available.

## Materials and Methods

### Gene family size calculation in model organisms.

We obtained gene information from the following sources: E. coli, E. coli Genome and Proteome Database (http://genprotec.mbl.edu/) (GenProtEC); *Saccharomyces cerevisiae,* Saccharomyces Genome Database (http://www.yeastgenome.org/) (SGD1.01); *Caenorhabditis elegans,* WormBase (http://www.wormbase.org/) (WB170); Drosophila melanogaster, Berkeley Drosophila Genome Project (http://www.fruitfly.org/) (BDGP 4.3); Homo sapiens, Ensembl Genome Database (NCBI36); Arabidopsis thaliana, Arabidopsis Information Resource (http://www.arabidopsis.org/) (TIR7.0). Then, based on the GenProtEC family annotation, 4,485 E. coli genes were grouped into 2,901 gene families (a singleton gene is counted as one family in our analysis); based on the Ensembl gene family annotation [29], 6,024 yeast genes were grouped into 4,661 families, 20,173 worm genes were grouped into 11,503 families, 14,116 fly genes were grouped into 9,477 families, and 22,357 human genes were grouped into 12,394 families. Thale cress gene families were classified using the MCL algorithm [30] with the default Ensembl parameters, which grouped 26,819 genes into 10,236 gene families. We excluded genes annotated with more than one gene family from our analysis.

### Computing the extent of protein under-wrapping.

For each of the six organisms under study, we constructed a set of non-redundant genes with at least one PDB representative structure. From the reported structure we calculated the extent of protein under-wrapping by determining the ratio of the number of insufficiently wrapped hydrogen bonds (dehydrons) to the total number of backbone hydrogen bonds in the structure. The dehydron identification from reported protein structure follows the protocol detailed in Chen *et al.* [12]. Together, our dataset includes 822 E. coli genes, 476 yeast genes, 29 worm genes, 94 fly genes, 2,275 human genes and 168 thale cress genes, for which we have both gene duplicability and protein structural data.

The extent of hydrogen-bond wrapping, ρ, measures the number of non-polar groups contained within a desolvation domain defined as two intersecting balls of fixed radius (∼thickness of three water layers) centered at the α-carbons of the residues paired by the amide-carbonyl hydrogen bond (Figure 1). In this study we adopted r = 5.7Å, and while the wrapping statistics on hydrogen bonds vary with this radius, the tails of the distribution remain invariant, thus enabling a unique identification of dehydrons. An across-PDB analysis reveals that hydrogen bonds are wrapped on average by ρ = 24.3 ± 4.8 non-polar groups for desolvation radius 5.7Å. Being insufficiently wrapped, dehydrons lie in the tails of the distribution, i.e., their desolvation microenvironment contains 19 or fewer non-polar groups, so that their ρ value is below the mean minus one Gaussian dispersion [12,15]. Thus, the overall under-wrapping of a protein is computed by determining the percentage of intramolecular hydrogen bonds with ρ ≤ 19. This criterion for identifying a dehydron fits the well-defined ansatz used to assess the wrapping statistics, which places dehydrons at the 8% percentile of most under-wrapped hydrogen bonds irrespective of the desolvation radius adopted [13–17]. Hence, the criterion is justified by the robustness of the results to variations in the assessment of the bond microenvironment.

The under-wrapping variation of a protein generated by structural differences in reported PDB entries is less than 8.8%. This variability arises from the different structural adaptations (induced fits) adopted by the protein in different crystallized complexes or from differences between uncomplexed protein structure in solution (often determined by NMR) and crystal structure. To account for such differences, the under-wrapping extent for each gene is typically averaged over all its PDB representations (Text S1). We obtained per-gene-family under-wrapping distributions by averaging the under-wrapping values among members within a gene family whenever available.

In this study, the wrapping computations involved more than 12,000 protein structures because a large fraction of the non-redundant proteins examined had various PDB representations with differences arising from the following sources: complexation diversity, level of structure resolution, NMR conformational diversity and high B-factors in the crystal (Text S1). The under-wrapping data obtained in our study are given in Tables S1–S6.

### Yeast WGD versus non-WGD duplicates analysis.

We obtained WGD gene duplicate pairs from Kellis et al. [22]. We used the Wilcoxon rank test (two-tailed) to determine whether the distributions of protein under-wrapping between WGD and non-WGD are different, since the underlying distributions are not normal. We used the GO term analysis tools [31] to map yeast genes into the GO terms in the default GO slim file.

## Supporting Information

Figure S1 Negative Correlations between Protein Under-Wrapping Extent and Gene Duplicability in Worm (A), Fly (B), and Thale Cress(C) (708 KB TIF)

Figure S2 Yeast Singletons Are More Under-Wrapped Than Duplicates in All the Functional Categories (654 KB TIF)

Table S1 E. coli Dataset (112 KB XLS)

Table S2 S. cerevisiae Dataset (69 KB XLS)

Table S3 C. elegans Dataset (19 KB XLS)

Table S4 D. melanogaster Dataset (27 KB XLS)

Table S5 H. sapiens Dataset (309 KB XLS)

Table S6 A. thaliana Dataset (33 KB XLS)

Text S1 Massive Protein Wrapping Computation (44 KB DOC)
