# Expression of Concern: Protein Under-Wrapping Causes Dosage Sensitivity and Decreases Gene Duplicability

**DOI:** 10.1371/journal.pgen.1005499

**Published:** 2015-09-14

**Authors:** The PLOS Genetics Editors

Concerns have been raised with regard to some of the data and the conclusions of the *PLOS Genetics* article, “Protein Under-Wrapping Causes Dosage Sensitivity and Decreases Gene Duplicability”. The authors have been contacted and an investigation into these concerns is ongoing.

This Expression of Concern should not be considered as a statement regarding the validity of the work, but rather as a notification to readers. *PLOS Genetics* will provide additional information as it becomes available.
