# Supplementary material for: Protein Under-Wrapping Causes Dosage Sensitivity and Decreases Gene Duplicability
Source: PLoS Genet. 2008 Jan 18;4(1):e11. doi: 10.1371/journal.pgen.0040011 (PMC2211539; doi:10.1371/journal.pgen.0040011)
Supplement: Supplementary file 1 [file pgen.0040011.sd001.doc]

**Supporting Text for:**

**Protein Under-wrapping Causes Dosage Sensitivity**

**and Decreases Gene Duplicability**

Han Liang, Kristina Rogale Plazonic, Jianping Chen, Wen-Hsiung Li and Ariel Fernández

**Massive wrapping computation**

The wrapping computation can be generically divided into two parts:

**Task 1)** Generation, curation and refinement of statistical ensembles of PDB-representations for each non-redundant PDB-reported protein with a computed gene duplicability index.

A PDB-representation is defined as a chain conformation for the protein specified in a PDB entry. Typically, NMR-generated entries provide many PDB-representations of a protein. For each of the six species studied, we first constructed a database of non-redundant PDB-reported proteins with computed gene duplicability indices. For each non-redundant protein, we then ran (in batch mode) an interrogator of the PDB in the "advanced query" model by entering its SwissProt ID to obtain a curated PDB-representation ensemble. Thus, our database was augmented to include all PDB-representations of each protein. This ensemble-based database dictates an averaging procedure needed to determine the under-wrapping value for each protein, which was performed at a later stage (Task 2).

Except for those proteins with only one PDB-representation, all PDB-representations in the ensemble for each protein were further classified into and weighted according to the following cases: a) there is more than one crystal structure for the isolated monomeric domain; b) the particular domain is crystallized in several different complexes with significant differences (as quantified by RMSD) in the induced fit of the domain of interest; c) the structure is reported from NMR data as an ensemble of conformations; d) the crystal B-factors (structural fluctuations) of the purported “wrapping” residues are large (>40).

Proteins with a singleton PDB-representation were directly used for the under-wrapping computation without any averaging schedule. Proteins with two or more complex PDB ensembles required an averaging procedure as specified below. For very under-wrapped proteins whose flexibility complicates structural characterization, the PDB ensemble is typically large (>20 PDB-representations), since NMR is often the spectroscopic method of choice. On the other hand, the ensemble tends to be small (<8 PDB-representations) for well-wrapped proteins. Correlation between under-wrapping and PDB-representativity was not attempted in view of the obvious bias of PDB towards relatively rigid structures that facilitate spectroscopic characterization. PDB-singletons are typically highly under-wrapped proteins that were nevertheless crystallized successfully, such as interleukin 1-alpha (PDB entry 2ILA, Gene: IL1A, SwissProt ID: P01583, under-wrapping: 49.22%).

The full curation/refinement for each non-redundant protein was performed as follows: a) Assignment of weights to PDB entries according to the level of structural resolution with three groups (low: ≥3.0Å, intermediate: >1.5Å but <3Å; high: ≤1.5Å, with weights 1, 2 and 3, respectively). b) If a protein presents different induced fits in different PDB-complexes, we included only as PDB representations the maximal group of induced fits with pairwise RMSD<5Å, thus avoiding averaging over widely dissimilar conformations. c) Parsing all conformations in each NMR PDB entry as separate PDB files of equal weight (i.e., 1). d) In the case of large B-factors (>40), the variability in the counting of wrapping groups may be large (as much as 33% of a particular ρ-value). Thus, structural refinement was performed in such cases (<1% of the non-redundant proteins considered) by running a 12ns-equilibration trajectory using the AMBER molecular dynamics package, adopting the reported PDB structure as the initial conformation and preparing the “refined” PDB file using the trajectory endpoint.

The process of construction of the ensemble-based PDB-derived databases for each species was considered complete once the statistical weights were assigned for each member of the ensemble according to the representation and quality of resolution and once all ambiguous cases (situation d) were resolved by structure refinement.

**Task 2)** Computation of protein under-wrapping values by performing an ensemble average for each non-redundant protein in our study.

The wrapping computation for a single PDB-representation was performed as described in the main text. The average under-wrapping for each protein was then obtained by adding the under-wrapping values for the PDB-representations, each multiplied by a factor of w/Ω, where w is the weight of the PDB-representation and Ω is the partition function for the ensemble defined as the sum of the weights for all PDB-representations for the protein.

To illustrate our calculation, two examples are shown below.

1. Gene ITSN2 (human intersectin 2, SwissProt ID Q9NZM3) has an ensemble obtained from 5 PDB entries. They are 1UDL, 1J3T, 1UE9, 1UFF and 1UHF. Since they were all obtained by NMR with 20 conformations in each entry, the size of the ensemble is 5 × 20 =1 00. Each conformation has the same weight w = 1; and the partition function Ω is 100. The ensemble average under-wrapping is 43.77% with a Gaussian dispersion of 5.28%.

2. Gene: IGF1R (Insulin-like growth factor 1 receptor kinase, SwissProt ID P08069) has 5 PDB representations all obtained by X-ray (resolution given in brackets): 1JQH (2.1 Å); 1D3A (2.1 Å); 1M7N (2.7 Å); 1P4O (1.5 Å) and 1IGR (2.6 Å). Therefore, the weights of these representations are 2, 2, 2, 3 and 2, respectively; and the partition function Ω = 2 + 2 + 2 + 3 + 2 = 11. The ensemble average under-wrapping is 39.04% with a Gaussian dispersion of 3.31%.

The sizes of the structural databases are as follows (the first number indicates the number of non-redundant PDB-reported proteins with computed gene duplicability indices, and the second number indicates approximately the number of PDB entries that were included in the ensembles according to cases a-d):

*E. coli*: 822, ~1,900 (TableS1)

*S. cerevisiae*: 476, ~1,100 (TableS2)

*C. elegans*: 29, ~60 (TableS3)

*D. melanogaster*: 94, ~300 (TableS4)

*H. sapiens*: 2275, ~9300 (TableS5)

*A. thaliana*: 168, ~200 (TableS6)
